# Molecular Mechanisms of Ischaemia-Reperfusion Injury and Regeneration in the Liver-Shock and Surgery-Associated Changes

**DOI:** 10.3390/ijms232112942

**Published:** 2022-10-26

**Authors:** Elise Pretzsch, Hanno Nieß, Najib Ben Khaled, Florian Bösch, Markus Guba, Jens Werner, Martin Angele, Irshad H. Chaudry

**Affiliations:** 1 Department of General, Visceral, and Transplant Surgery, Ludwig-Maximilians-University Munich, 81377 Munich, Germany; 2 Department of Medicine II, University Hospital, LMU Munich, 81377 Munich, Germany; 3 Department of General, Visceral and Pediatric Surgery, University Medical Center Goettingen, 37075 Goettingen, Germany; 4 Department of Surgery, University of Alabama at Birmingham, Birmingham, AL 35294, USA

**Keywords:** ischaemia-reperfusion, liver, liver surgery

## Abstract

Hepatic ischemia-reperfusion injury (IRI) represents a major challenge during liver surgery, liver preservation for transplantation, and can cause hemorrhagic shock with severe hypoxemia and trauma. The reduction of blood supply with a concomitant deficit in oxygen delivery initiates various molecular mechanisms involving the innate and adaptive immune response, alterations in gene transcription, induction of cell death programs, and changes in metabolic state and vascular function. Hepatic IRI is a major cause of morbidity and mortality, and is associated with an increased risk for tumor growth and recurrence after oncologic surgery for primary and secondary hepatobiliary malignancies. Therapeutic strategies to prevent or treat hepatic IRI have been investigated in animal models but, for the most part, have failed to provide a protective effect in a clinical setting. This review focuses on the molecular mechanisms underlying hepatic IRI and regeneration, as well as its clinical implications. A better understanding of this complex and highly dynamic process may allow for the development of innovative therapeutic approaches and optimize patient outcomes.

## 1. Introduction

Ischemia reperfusion injury (IRI) is a complex and dynamic process that consists of two phases: a cessation of blood supply to an organ (e.g., due to arterial occlusion) leading to a shortage of oxygen and an imbalance of metabolic supply and demand. As opposed to ischemia occurring with a total lack of blood flow, hypoxia may be caused due to a restriction/reduction of blood flow that occurs with trauma-hemorrhages. The reperfusion following ischemia is characterized by a restoration of blood flow associated with a paradox exacerbation of tissue injury due to the previous lack of oxygen, nutrient delivery, and buildup of metabolic by-products [1,2].

Hepatic IRI can be due to a regional cessation of blood supply during liver surgery (liver resection, transplantation) or, in significantly less cases, due to a systemic reduction of perfusion during shock and trauma. In the latter case, small areas of regional ischemia may occur following trauma and major blood loss in order to preserve blood flow to vital organs. However, if uncorrected, those areas produce similar responses as organ ischemia in the long term, and lead to tissue and organ damage [3]. Hepatic IRI is a major cause of functional liver failure (liver resection, shock) and graft dysfunction (transplantation) and, if severe enough, can result in systemic inflammatory response syndrome (SIRS) or lead to multiple organ failure [4]. In this respect, hepatic IRI represents a major complication in liver surgery and shock, and is associated with increased postoperative morbidity, mortality, and other worse outcomes [4,5]. Furthermore, hepatic IRI is associated with tumor progression and metastasis following liver resections due to an oncological cause [6].

In order to prevent liver dysfunction, attenuate postoperative morbidity, decrease mortality, reduce the likelihood of tumor recurrence due to hepatic IRI, and therapeutically target liver dysfunction, it is pivotal to understand the underlying mechanisms of this complex process.

This review elaborates on the molecular mechanisms of hepatic IRI and regeneration following liver surgery and shock that could be targeted by potential therapeutic strategies to prevent or attenuate hepatic IRI. As there are various mechanisms and signaling pathways that have been investigated in previous studies, this article focuses on mechanisms that have been demonstrated to have the highest relevance in preclinical models, that may be most relevant from a translational standpoint, and might have clinical implications in the near future.

## 2. Molecular Mechanisms and Pathophysiology of IRI

### 2.1. Anaerobic Metabolism, Acidosis, and ATP Depletion

Liver ischemia, whether caused by a regional or systemic cessation of blood flow, leads to a variety of alterations in cellular function. The decreased availability of oxygen to the tissue places an increased demand on anaerobic metabolism that results in a shift from an aerobic to an anaerobic metabolic state associated with a depletion in ATP (adenosine triphosphate) levels [7]. Simultaneously, anaerobic glycolysis is accelerated, leading to an increased release of lactic acid, an accumulation of oxygen radicals, and a disequilibrium in H^+^, Na^+^, and Ca^2+^ homeostasis [8,9,10,11]. Lactic acid has been linked to cellular dysfunctions and impaired signaling interactions, including altered macrophage responses and depressed antigen-presentation by Kupffer cells associated with early immunosuppression after shock or extensive surgery [12,13]. Similarly, an overload in cellular calcium levels has been suggested to contribute to the suppression of cellular immunity, potentially by impairing macrophage antigen presentation function [12]. In addition, increased intracellular calcium levels have been shown to activate Ca^2+^-dependent enzymes, such as protein kinase C and phospholipase C, resulting in apoptosis and cell death [14]. Further consequences of the accumulation of toxic acidic metabolites include mitochondrial hypofunction and damage, microcirculatory dysfunction, and cell injury, and will be further elucidated below [15].

### 2.2. Mitochondria, Oxidative Stress and Reactive Oxygen Species

Reperfusion of the liver, associated with a concomitant reintroduction of oxygen to ischemic/severely hypoxic tissue, leads to a significant increase in the production of free radicals, such as reactive oxygen (ROS) and reactive nitrogen species. Mainly produced by mitochondria, they exceed the amount produced during the ischemic phase, which is due to the fact that the immediate increase in oxygen supply exceeds the rate of the anaerobic metabolism returning to an aerobic state [10,16]. Insights from transgenic knockout models suggest that the source of ROS production changes during different phases of IRI; whereas, Kupffer cells are the main source of ROS in the early phase, natural killer T cells take over in a later phase, with neutrophils generating the main amount in very late stages [15]. These mitochondria-generated ROS have detrimental effects on enzymes, proteins, nucleic acids, and the cytoskeleton, thereby leading to mitochondrial dysfunction and damage. Furthermore, ROS lead to an upregulation of Tumor Necrosis Factor-α (TNF-α) and the transcription factor Nuclear Factor κB (NF-κB) with an increased release of downstream molecules, such as interleukins (e.g., IL-1), thereby indirectly causing cellular injury [17]. The cellular damage is not limited to hepatocytes, but also includes endothelial cells, which results in microcirculatory dysfunction due to the destruction of the microvascular integrity [16,18,19].

### 2.3. Microcirculatory Dysfunction

After reperfusion, microvascular dysfunction is among the earliest changes and is characterized by vasoconstriction, the destruction of the sinusoidal endothelial cell (SEC) barrier, expression of adhesion molecules, and an accumulation of leukocytes, including neutrophils in the liver. Within two hours of reperfusion, Kupffer cells release proinflammatory cytokines (e.g., TNF-α, interleukin-1β (IL-1β), interleukin-6 (IL-6)) which leads to an activation of sinusoidal endothelial cells and an imbalance of nitric oxide (NO) production from NO synthase and endothelin-1 (ET-1), which causes vasoconstriction [20,21,22,23]. The activation of SECs is followed by an upregulation of adhesion molecules (e.g., intercellular adhesion molecule 1 (ICAM-1), vascular cell adhesion molecule 1 (VCAM-1)), and integrins (e.g., CD11b/CD18). Attracted by the respective cytokines, neutrophils in the blood stream attach to the adhesion molecules and transmigrate through the impaired sinusoidal endothelial barrier, thereby exiting the vasculature towards the site of the liver damage [24,25]. The infiltration of neutrophils further impairs hepatic perfusion and has deleterious effects on hepatocytes due to the secretion of toxic proteases (e.g., cathepsin G, matrix-metalloproteases) resulting in further hepatic liver injury [26,27].

### 2.4. Pro- and Anti-Inflammatory Cytokines

As mentioned above, activated Kupffer cells in early stages and neutrophils in late stages secrete IL-1 and TNF-α, which leads to an activation of various downstream pathways inducing an inflammatory response, thereby contributing to hepatic IRI. In this respect, downstream targets of TNF-α include NF-κB, mitogen-activated protein kinase, and c-Jun N-terminal kinase (JNK) [28]. Moreover, TNF-α enhances the upregulation of the previously mentioned adhesion molecules ICAM-1 and VCAM-1, thereby facilitating neutrophil accumulation [29]. Other cytokines involved in IRI include interleukins, such as IL-6, IL-12, IL-23, vascular endothelial growth factor (VEGF), and hepatocyte growth factor (HGF) [30]. IL-12 and IL-23 stimulate CD4 T cells to release IL-17, which serves as another attractant for neutrophils in the liver, thereby aggravating hepatic injury [31,32]. Additionally, they promote TNF-α production via NF-ĸB activation, and IL-23 activates the IFN-γ/IRF-1 pathway [32]. Conversely, TNF-α, IL-1, IL-12, and IL-23 function as proinflammatory cytokines followed by immunosuppression contributing to liver damage; IL-6, VEGF, and HGF seem to act the opposite way contributing to liver regeneration. In this respect, IL-6 reduces liver damage and promotes hepatocyte proliferation by enhancing glutathione expression, activating STAT3 signaling, and eliminating reactive oxygen species [33]. Interestingly, VEGF seems to have a dual role in IRI: while endogenous VEGF is expressed and functional to initiate hepatic IRI, exogenous VEGF has a beneficial effect and contributes to the regeneration of the post-ischemic liver [34]. HGF also seems to have protective functions and has been shown to attenuate IRI by suppressing oxidative stress, promoting hepatocyte proliferation through activation of ERK1/2 signaling, enhancing glutathione expression, and downregulating ICAM-1, thereby impeding neutrophil adhesion and transmigration into the liver [35,36,37].

In summary, IRI due to surgical trauma or shock leads to an inflammatory response characterized by an increased release of pro-inflammatory cytokines. After reperfusion, this state of inflammation is followed by a counter-regulatory anti-inflammatory response with a rise in anti-inflammatory cytokines, while pro-inflammatory cytokine levels may simultaneously remain high. In this respect, IRI leads to a highly vulnerable state with a concurrent expression of pro- and anti-inflammatory cytokines that predominate in an alternating manner [7]. The local inflammatory state of the injured tissue can be followed by a systemic inflammatory response, leading to the systemic inflammatory response syndrome (SIRS) and even multiple organ failure. The anti-inflammatory response has been shown to induce an immunosuppressive state that can even favor tumor growth and metastasis in the liver remnant after hepatic surgery via an activation of cell invasion and migration pathways [4,6,12,38,39]. In this respect, the processes described that start as a local reaction within injured tissue can have “global” consequences involving initially healthy organs.

### 2.5. NF-κB

NF-ĸB is a transcription factor that plays a key role in mediating inflammatory gene expression during IRI. Activated by hypoxia or cytokines, NF-ĸB translocates to the nucleus of cells, binds to DNA, and induces the transcription of respective target genes. As elucidated above, NF-ĸB regulates gene expression in various cells during IRI. As such, NF-ĸB promotes the expression of TNF-α in hepatocytes, the expression of TNF-α and IL-6 in Kupffer cells, and the expression of adhesion molecules ICAM-1 and VCAM-1 in endothelial cells [40,41,42]. Interestingly, NF-ĸB has a dual role in IRI: while it promotes the pro-inflammatory state during the acute injury phase, it also exhibits hepatoprotective functions and fosters regeneration after injury [43,44].

### 2.6. Apoptosis and Necrosis

Hepatic reperfusion is not only characterized by an interplay of pro- and anti-inflammatory mediators, but also by massive cell injury mainly affecting hepatocytes and sinusoidal endothelial cells [15]. It remains controversial as to whether this is due to apoptosis via activation of caspase-3 and caspase-8 by TNF-α or necrosis. However, various studies suggest that both pathways might coexist, sharing mechanistic pathways [27,45,46].

### 2.7. miRNAs

miRNAs are a subset of short single-chain, non-coding RNAs that regulate gene expression, and can thereby modulate IRI on a posttranscriptional and posttranslational level [47]. In this respect, a variety of miRNAs have been identified to impair or ameliorate the state of hepatic IRI, and therefore have been suggested to serve as biomarkers for early diagnosis of IRI as a reflection of the present state of IRI. miRNAs that have been shown to impair the condition of hepatic IRI, when upregulated, include miRNA-223, miRNA-191, miRNA-133a-5p, miRNA-17, miRNA-450-5p, and others. Respective targets are STAT3, NF-κB, IL-1, and TNF-α that have been previously described to be pivotal mediators during IRI [48,49,50,51,52]. Other miRNAs that worsen the condition of IRI, when downregulated, are miRNA-142-3p, miRNA-146a, miRNA-200c, and miRNA-34a targeting factors, such as JNK, ZEB1, and p53 [53,54,55,56,57]. In contrast, there are also miRNAs that ameliorate the condition of IRI, among which are miRNA-370, miRNA-182-5p, miRNA-125b (when upregulated), and miRNA-128-3p (when downregulated) by targeting toll-like receptor 4 (TLR4), TNF-α, IL-1, IL-6, and NF-κB, [58,59,60,61,62]. In this respect, miRNAs worsen or attenuate IRI by modulating inflammatory cell responses, oxidative stress reactions and apoptosis, and cell death, processes that have been elaborated on in previous paragraphs.

### 2.8. Innate and Adaptive Immune Activation

**Figure 1:**
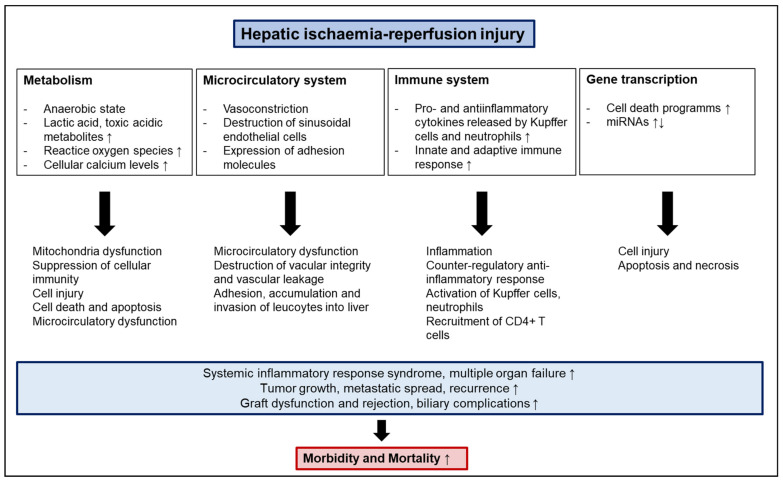
Components of hepatic ischemia reperfusion injury.

Both innate and adaptive immune responses play an important part in IRI [63]. The abovementioned cell death, as well as mitochondria dysfunction, induce the production of danger-associated molecular patterns (DAMPs), such as high-mobility group box 1 (HMGB1), that trigger the innate immune response. In this context, toll-like receptors (TLR) such as TLR 4, 7, and 9, as well as nucleotide-binding oligomerization domain (NOD)2, have been shown to promote IRI [64]. TLRs are expressed on various liver cell types, including Kupffer cells, hepatocytes, and dendritic cells. They recognize DAMPs and propagate the inflammatory response. After reperfusion, HMGB1 levels increase and bind to TLR4, leading to an upregulation of downstream pro-inflammatory pathways. However, TLRs have also been shown to alleviate the IRI. In this respect, a lack of intestinal TLR 9 has been demonstrated to increase apoptosis and inflammation, thereby aggravating IRI [65]. CD4+ T-lymphocytes seem to play a major role during adaptive immune responses. Trapped in the constricted sinusoids, they produce granulocyte-macrophage colony-stimulating factor (GM-CSF), interferon gamma (IFN-γ), and TNF-β, which amplify cytokine release and the activation of Kupffer cells [66,67]. Kupffer cells are tissue-fixed macrophages of the liver and are in bidirectional crosstalk with CD4+ T-cells [68,69]. In this respect, Kupffer cells release ROS, TNF-α, and IL-1, thereby triggering the recruitment of CD4+ T-cells, as well as the activation of sinusoidal endothelial cells. In turn, CD4+ T-cells can activate Kupffer cells, thereby promoting further inflammation [68]. However, Kupffer cells might have a dual role in IRI. In this respect, Kupffer cells can release IL-10, an anti-inflammatory cytokine that can suppress NF-κB activation and inhibit the expression of pro-inflammatory factors, such as TNF-α, IL-1β, IFN-γ, IL-2, and adhesion molecules, such as ICAM-1 [70]. Furthermore, CD4+ T-cells help recruit neutrophils to the site of injury through the release of IL-17. Alternatively, depletion of CD4+ T-cells in mice has been shown to be associated with a decrease in neutrophil recruitment [71]. Treg cells have also been demonstrated to be involved in the adaptive immune response, potentially exhibiting a similar protective function. In this context, CD4+CD25+Foxp3+ Treg cells have been shown to attenuate IRI via the PI3K/Akt/mTOR signaling pathway [72,73]. Overall, both innate and adaptive immune responses participate in IRI, crosstalk with each other, and function in partially synergistic or counteractive ways (all components of IRI are summarized in Figure 1).

## 3. Cold Ischemia Versus Warm Ischemia Reperfusion Injury

Hepatic IRI can be categorized into two types: cold ischemia and warm ischemia. While the previous paragraphs have focused on the molecular mechanisms underlying hepatic IRI in general, the majority of which is present in both types of IRI, this section aims to elucidate the main differences between cold and warm ischemia.

Cold storage ischemia occurs during organ preservation before liver transplantation. Warm ischemia occurs in the setting of transplantation, trauma and shock, and elective liver surgery (e.g., hepatic resections) when hepatic blood supply and oxygen delivery is temporarily interrupted.

### 3.1. Cold Ischemia

Cold IRI is characterized by massive damage to sinusoidal endothelial cells, a destruction of the endothelial integrity, and microcirculatory dysfunction. Studies have shown that endothelial cells are more sensitive to cold storage than hepatocytes, which results in higher cell death rates through apoptosis and necrosis of endothelial cells compared to liver parenchymal cells [74,75,76]. Endothelial cell damage is followed by Kupffer cell activation, leading to an increased release of cytokines and ROS as previously described. In this respect, Kupffer cell activation has been demonstrated to correlate and increase with increasing cold storage times [75].

### 3.2. Warm Ischemia

In contrast to cold ischemia, warm IRI is dominated by hepatocellular damage. In this regard, hepatocytes have been shown to be more sensitive to warm IRI (37 °C), while sinusoidal endothelial cells are more susceptible to cold IRI (4 °C) [15]. Warm IRI can be categorized into two phases: the early phase starts within two hours after reperfusion and is characterized by oxidative stress and an activation of immune cells. The late phase starts within 6 to 48 h after reperfusion and is characterized by the accumulation of neutrophils and hepatocellular injury [27,77,78,79]. However, the underlying processes are intertwined and overlap both phases (for more details, please see previous paragraphs).

## 4. Liver Regeneration after IRI

**Figure 2:**
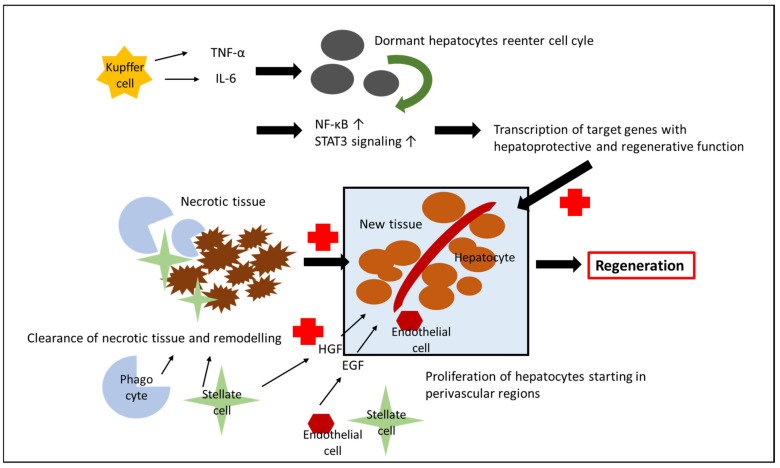
**Mechanisms of liver regeneration after hepatic IRI**. Kupffer cells release TNFα and IL6 stimulating dormant hepatocytes to reenter the cell-cycle. STAT3 signaling is activated, leading to the transcription of hepatoprotective target genes that promote hepatocyte survival and proliferation. The necrotic liver tissue is cleared by, e.g., phagocytes and stellate cells. Extracellular matrix, stellate, and endothelial cells release HGF and EGF which stimulates hepatocyte proliferation starting in perivascular regions resulting in liver regeneration.

The liver is unique in its capacity to regenerate after injury, a process that is mediated by cytokines, growth factors, and a supportive microenvironment [80]. In general, after uncomplicated hepatic resections, TNF-α and IL-6, which are released from Kupffer cells, prime viable hepatocytes to reenter the cell cycle and initiate NF-κB activation and STAT3 signaling, resulting in the transcription of target genes that are essential for liver regeneration. Subsequently, after reentering the cell-cycle, hepatocytes proliferate, a process that is further promoted by HGF, which is released from the extracellular matrix, hepatic stellate cells and endothelial cells after liver resection, and epidermal growth factor [81,82]. As opposed to uncomplicated liver resections, hepatocytes are stressed after IRI but the stimulus for their proliferation remains unknown [83,84]. During the regeneration process, which begins in perivascular regions, phagocytes, stellate cells, and other nonparenchymal cells clear the necrotic tissue and remodel injured areas so that proliferating hepatocytes can restore the normal architecture, replace the unfunctional liver mass, and generate new functional mass [83,84]. In this regard, NF-ĸB, which has been described in previous paragraphs to not only promote the pro-inflammatory state, but also exert hepatoprotective and regenerative functions, plays an important role in fostering the regenerative potential of hepatocytes [43,44]. Similarly, cytokines (e.g., ELR^+^ CXC chemokines, such as IL-1) that promote the inflammatory state during the acute injury phase now impact the hepatocytes in an opposite way and promote regeneration, a mechanism that is most likely due to different concentrations of cytokines; while high concentrations seem to induce hepatocyte damage, low concentrations promote hepatocyte regeneration [85] (Figure 2).

## 5. Clinical Implications of IRI in Different Settings

While the previous paragraphs focused on the molecular mechanisms of IRI during the acute phase and regeneration, this chapter discusses the long-term impact of inflammation induced by IRI on the liver.

### 5.1. Tumor Growth, Metastasis, and Recurrence

Various studies in both humans and animals have shown that IRI is a strong stimulus for tumor growth, metastasis, and recurrence [6,86,87,88]. During hepatic surgery, hemorrhage and shock are common, leading to IRI due to a systemic reduction in blood perfusion, and have been shown to promote tumor growth and recurrence. In order to control hepatic bleeding, vascular clamping techniques are applied; however, these by themselves can cause hepatic IRI due to a regional obstruction of perfusion and oxygen supply. Overall, the surgical stress due to hemorrhage or vascular clamping and acute phase of IRI with an excess expression of pro-inflammatory cytokines, reactive oxygen species, and adhesion molecules, as well as matrix-metalloproteases (e.g., MMP9), promote tumor cell adhesion, migration, invasion (e.g., via c-MET), and angiogenesis [6,7,86,89,90]. In a colon cancer rat model, hepatic IRI resulted in an increase in liver metastases due to the upregulation of the adhesion molecule E-selectin (and respective E-selectin mRNA). While the amount of liver metastases was time-dependent, with a ten times higher amount of tumor nodules in the 60 (versus 30) minutes ischemia group, metastatic dissemination was present in both clamped and unclamped lobes pointing towards the systemic impact of IRI [86]. Another study investigating colorectal cancer in xenograft models demonstrated that hepatic IRI due to vascular clamping accelerated the outgrowth of pre-existent hepatic micrometastases, resulting in a worse prognosis. However, accelerated tumor growth and tissue necrosis were effectively prevented by intermittently occluding blood flow. As opposed to the previously mentioned study by Doi et al., tumor growth was only stimulated in the clamped lobes [87]. In summary, whether tumor growth after hepatic IRI is due to locally operating mechanisms affecting the clamped lobe and factors potentially disseminating to the unclamped lobe, or whether it is due to the involvement of systemically released factors remains controversial, has not been yet elucidated. Man et al. demonstrated that hepatic IRI of the small liver remnant after major hepatectomy for colorectal liver metastases in mice promoted further metastatic spread to the liver and lungs, due to an upregulation of mRNA levels for Cdc42, ROCK (Rho kinase), vascular endothelial growth factor, and an activation of hepatic stellate cells, all leading to an activation of cell adhesion, invasion, and angiogenesis [6]. Subsequently, ischemia of the liver remnant in patients after resection of colorectal cancer liver metastases was associated with worse cancer-specific and recurrence-free survival [88]. A recent meta-analysis assessing the impact of liver pedicle clamping in patients receiving liver surgery for hepatocellular carcinoma, reported significantly shorter overall survival and higher tumor recurrence rates in patients with pedicle clamping versus the control group [91]. However, prevention of tissue hypoxia through targeting the HIF-1α pathway and improvement of microcirculatory flow might limit recurrence after liver surgery [92].

### 5.2. Graft Fibrosis

Liver regeneration following hepatic IRI is associated with prolonged fibrosis, especially at the interface between necrotic tissue and regenerating liver due to an increased expression of profibrotic genes in the ischemic liver. In this respect, collagen deposition is enhanced along the injured sites and an increased infiltration of α-smooth muscle actin positive hepatic stellate cells/myofibroblast can be observed [37,93]. However, after full regeneration and restoration of functional liver mass, which takes several weeks, the fibrosis seems to be self-limiting and disappears [94]. In fact, a recent study showed that the fibrotic liver, despite having a higher degree of injury, recovered more quickly than normal liver after hepatic IRI, which was attributed to the increased numbers of hepatic stellate cells, hepatic progenitors cells, and macrophages to the fibrotic sites [93].

### 5.3. Biliary Complications and Graft Dysfunction

Biliary complications are among the most frequent complications after transplantation, and can lead to graft dysfunction or loss. Additionally, they have been associated with increased morbidity and mortality rates [95]. Hepatic IRI can cause intra- and extrahepatic nonanastomotic biliary strictures after transplantation, a process that is the result of direct injury of cholangiocytes and damage of peribiliary vascular regions leading to apoptosis and necrosis of cholangiocytes [96]. Moreover, direct immunologic cellular injury to the bile duct epithelium is the major underlying cause for tissue damage during liver allograft rejection. The exact mechanisms underlying this process are beyond the theme of this article and are discussed elsewhere [97].

## 6. Therapeutic Approaches

There are a variety of therapeutic approaches with the aim to attenuate or prevent hepatic IRI, including surgical and pharmacological strategies and the application of perfusion machines [11,98,99,100]. Overall, the therapeutic measures investigated all aimed to target the abovementioned underlying molecular processes of hepatic IRI. These include the reduction of reactive oxygen species by administration of free radical scavengers (e.g., N-acetyl-cysteine), the modulation of the cytokine response and blockage of immune activation (e.g., steroids, gadolinium chloride), and the preservation of organ function through ex vivo organ perfusion and ischemic preconditioning; however, many approaches have only been validated in preclinical studies, or have not been proven successful in the clinical setting underscoring the complexity of IRI [11,98,101,102]. The effect of immunosuppressants (azathioprine, cyclosporine A, tacrolimus, rapamycin, etc.) has been investigated in previous studies, which have been shown to be of a protective nature [103,104,105]. In this respect, immunosuppressants can attenuate IRI of the liver through the reduction of neutrophil and leukocyte accumulation, and modification of the early-local immune response by decreasing the ratio of proinflammatory to anti-inflammatory T lymphocytes and increasing the Treg proportion; however, they cannot fully prevent hepatic IRI. In this respect, additional therapies are required that have the ability to not only eliminate the effector cells and products, but instead harness lymphoid subpopulations (e.g., Treg cells) with regulatory capability to modify the immune response [105]. However, to our knowledge, these strategies have not been tested beyond preclinical studies. From a surgical standpoint, there are a few considerations that can help to minimize hepatic IRI and the respective consequences. As previously described, intraoperative hemorrhage is a common complication during extended liver resections and has been shown to be associated with increased postoperative morbidity, increased tumor recurrence, and worse prognosis [7]. Therefore, various vascular occlusion techniques (e.g., Pringle Maneuver) have been investigated in order to reduce blood loss. However, at the same time, vascular clamping by itself aggravates hepatic IRI; also contributing to postoperative morbidity and worse oncologic outcomes. In this respect, a balance between tolerated blood loss and restriction of hepatic blood flow is required. The ideal clamping time is still under debate; however, intermittent clamping with 30 min of restricted hepatic flow and 5 min of reperfusion seems to be an effective medium [100]. According to a systematic review by Hoekstra et al., there is no evidence supporting further strategies to attenuate hepatic IRI, such as ischemic preconditioning or intraoperative pharmacological interventions [100]. Lastly, it needs to be highlighted that, apart from heavy intraoperative blood loss or vascular clamping, ischemia of the liver remnant after extended liver resections can be due to an additional cause: the presence of insufficiently or nonperfused liver tissue due to imprecise liver resections or due to excessive resections with unintentional damage to a segment’s inflow or out-flow vessels. However, such a factor can be positively influenced by meticulous operative techniques by liver surgeons [88,99].

## 7. Future Directions

With the aim to attenuate liver damage due to hepatic IRI and decrease morbidity, as well as mortality, future strategies should include studies on IRI prevention not only focusing on the phases of ischemia and reperfusion, but also regeneration after tissue damage. As indicated above, hepatic IRI can be due to regional cessation of blood flow, but could also result from a systemic reduction in blood supply. Thus, recognizing that the liver is only a part of a multi-organ system, future studies may wish to approach hepatic IRI in a more holistic manner. Starting points for further studies could include the following: targeting macrophages and neutrophils to impose reparative phenotypes [106], harnessing lymphoid subpopulations (e.g., Treg cells) with regulatory capability to modify the immune response [105], application of stem cells to restore functional and structural components of the liver [107], and also clinical features, such as pre-operative exercise-based therapy to induce anti-inflammatory trained immunity [108]. Moreover, as the majority of studies are of experimental preclinical nature, translating the results into the clinical arena is required, and might be of immense benefit to improve patient outcomes.

## 8. Conclusions

Hepatic IRI occurs in a variety of settings, including liver surgery (hepatic resections, liver transplantation), liver preservation for transplantation, trauma, and hemorrhagic shock. Not only does it have adverse effects on morbidity (e.g., graft dysfunction, biliary complications), but it is also associated with increased mortality, as well as metastatic spread and recurrence in cancer patients. The molecular mechanisms of hepatic IRI and regeneration are complex and highly dynamic, and involve a variety of signaling cascades and downstream mediators modulating the cellular metabolic state, the immune response, gene transcription, and microvascular function. While various therapeutic approaches have been investigated in animal models, there is a need for validation in clinical studies to effectively treat and prevent hepatic IRI in the future to optimize patient outcomes.

## Data Availability

Not applicable.
